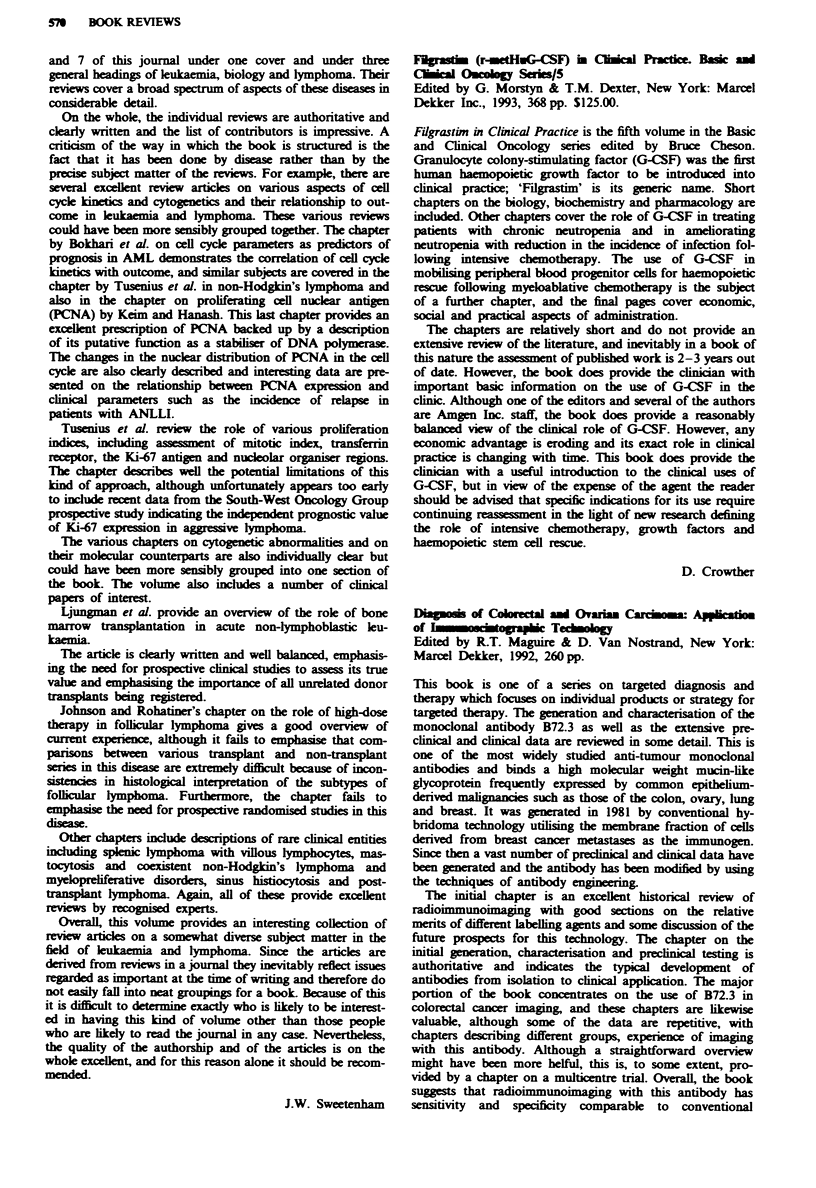# Filgrastim (r-metHuG-CSF) in clinical practice. Basic and clinical oncology series/5

**Published:** 1994-09

**Authors:** D. Crowther


**Filgrastim (r-metHuG-CSF) in Clinical Practice. Basic and Clinical Oncology Series/5**

Edited by G. Morstyn & T.M. Dexter, New York: Marcel Dekker Inc., 1993, 368 pp. $125.00.

*Filgrastim in Clinical Practice* is the fifth volume in the Basic and Clinical Oncology series edited by Bruce Cheson. Granulocyte colony-stimulating factor (G-CSF) was the first human haemopoietic growth factor to be introduced into clinical practice; 'Filgrastim' is its generic name. Short chapters on the biology, biochemistry and pharmacology are included. Other chapters cover the role of G-CSF in treating patients with chronic neutropenia and in ameliorating neutropenia with reduction in the incidence of infection following intensive chemotherapy. The use of G-CSF in mobilising peripheral blood progenitor cells for haemopoietic rescue following myeloablative chemotherapy is the subject of a further chapter, and the final pages cover economic, social and practical aspects of administration.

The chapters are relatively short and do not provide an extensive review of the literature, and inevitably in a book of this nature the assessment of published work is 2–3 years out of date. However, the book does provide the clinician with important basic information on the use of G-CSF in the clinic. Although one of the editors and several of the authors are Amgen Inc. staff, the book does provide a reasonably balanced view of the clinical role of G-CSF. However, any economic advantage is eroding and its exact role in clinical practice is changing with time. This book does provide the clinician with a useful introduction to the clinical uses of G-CSF, but in view of the expense of the agent the reader should be advised that specific indications for its use require continuing reassessment in the light of new research defining the role of intensive chemotherapy, growth factors and haemopoietic stem cell rescue.

D. Crowther